# Clotting Factors in COVID-19: Epidemiological Association and Prognostic Values in Different Clinical Presentations in an Italian Cohort

**DOI:** 10.3390/jcm9051371

**Published:** 2020-05-07

**Authors:** Pierpaolo Di Micco, Vincenzo Russo, Novella Carannante, Michele Imparato, Stefano Rodolfi, Giuseppe Cardillo, Corrado Lodigiani

**Affiliations:** 1 Internal Medicine Unit, Fatebenefratelli Hospital, 80123 Naples, Italy; michele.imparato@fatebenefratelli.it; 2 Cardiology Unit, Department of Translational Medical Sciences, University of Campania “Luigi Vanvitelli”—Monaldi Hospital, piazzale Ettore Ruggeri, 80131 Naples, Italy; 3 First Division of Infectious Diseases, Cotugno Hospital, 80131 Naples, Italy; carannantenovella@gmail.com; 4 Department of Medical Sciences, Humanitas University, 20090 Milan, Italy; stefano.rodolfi@humanitas.it (S.R.); corrado.lodigiani@humanitas.it (C.L.); 5 Medylab, Biochimica avanzata Laboratory, 81030 Lusciano (CE), Italy; giuseppe.cardillo.75@gmail.com; 6 Center for Thrombosis and Hemorrhagic Diseases, Humanitas Clinical and Research Hospital, Rozzano, 20089 Milan, Italy

**Keywords:** COVID-19, SARS-CoV2, alteration of hemostasis, disseminated intravascular coagulation, fibrinogen, prothrombin time

## Abstract

Introduction: A novel highly pathogenic human coronavirus able to induce severe acute respiratory syndrome (SARS) has been recently recognized as the cause of the coronavirus disease 2019 (COVID-19) outbreak, which has spread rapidly from China to other countries. Little is known about laboratory prognostic markers in COVID-19 patients. The aim of our study was to describe the basic clotting parameters in COVID-19 patients and their prognostic role in different clinical forms of the disease. Material and Methods: We enrolled 67 COVID-19 patients admitted to the Emergency Department. A cohort of 67 age- and sex-matched non-COVID-19 patients with acute respiratory illness was used as a control group. For all patients, platelet count (PLT), prothrombin time (PT), activated thromboplastin time (aPTT), C-reactive protein (PCR), fibrinogen, and D-dimer were determined. The COVID-19 population was divided in two groups according to the presence or absence of SARS. The clotting factors values were compared between the groups. Results: At admission, the COVID-19 patients showed statistically significant increased levels of fibrinogen (601.5 (480–747) vs. 455 (352.5–588.5) mg/dL; *p* = 0.0000064), and a higher percentage of patients had fibrinogen levels >400 mg/dL (86% vs.58%; *p* = 0.0054) compared to the control group. The levels of fibrinogen were higher in COVID-19 patients with SARS compared to those without SARS (747 (600.0–834.0) vs. 567 (472.5–644.50); *p* = 0.0003). Conclusion: Fibrinogen seems to increase early in COVID-19 patients and may be used as a risk stratification marker for the early detection of a subgroup of COVID-19 patient at increased risk to develop SARS, who might benefit from a different and thorough clinical surveillance and treatment.

## 1. Introduction

A novel highly pathogenic human coronavirus able to induce severe acute respiratory syndrome (SARS) has been recently recognized in Wuhan, China, as the cause of the coronavirus disease 2019 (COVID-19) outbreak which has spread rapidly from China to other countries, causing a pandemic with alarming morbidity and mortality related to its possible severe clinical presentation, namely, severe acute respiratory syndrome coronavirus 2 (SARS-CoV-2) [1,2].

The pathogenesis of SARS in COVID-19 patients seems to be particularly complex; however, data so far available suggest that SARS-CoV 2 infection may lead to a dysregulated immune response with increased interleukin 6 (IL-6) levels, which is responsible forprogressive lung injury and bilateral multifocal interstitial pneumonia [3].

To date, Italy is one of the hardest hit countries by the COVID-19 pandemic after China, with about 172,000 laboratory-confirmed cases [4]. Little is still known about the laboratory prognostic markers in COVID-19 patients and how they may help to identify patients at increased risk to evolve toward a worse clinical condition [5,6]. In this respect, no European cohort study has been published yet. The aim of our study was to describe the basic clotting parameters in COVID-19 patients and their prognostic role in different clinical forms of the disease.

## 2. Materials and Methods

From a large cohort of 100 patients admitted from February 2020 to March 2020 for fever and dyspneato the Emergency Departments (ED) of three Italian Hospitals (Humanitas Hospital of Milan, Cotugno Hospital of Naples, Fatebenefratelli Hospital of Naples), we enrolled 67 patients with confirmed diagnosis of COVID-19. An age- and sex-matched cohort of 67 patients with non-COVID-19 acute respiratory illness admitted to the ED was used as a control group. At admission, all patients underwent medical history recording, physical examination, laboratory evaluation. Laboratory tests includedthe determination of platelet count (PLT), prothrombin time (PT) expressed in seconds, activated thromboplastin time (aPTT) expressed as a ratio, C-reactive protein (PCR), fibrinogen, and D-dimer. Chest X-Ray and/or Computed Tomography (CT) scan were also performed to rule out pneumonia in one or multiple sites.

The COVID-19population was divided in two groups according to the diagnosis of isolated pneumonia or pneumonia withSARS. The clotting factors values werecompared between these two groups.

The institutional ethics committee approved the protocol (FBGID-90320). Verbal and written informed consent for participation was provided by all patients.

### Statistical Analysis

The Anderson–Darling test was used to analyze data normality. Continuous variables were reported using the median and interquartile intervals. Categorical variables were indicated as frequency counts and percentages. Differences between groups were evaluated using the 2-tailed Fligner–Policello/Fligner–Killeen test for continuous data and the Barnard’s or Fisher’s test for categorical variables. Furthermore, in order to find differences between the pneumonia group and the control group, as well as between the SARS group and the control group, the Dunn’s test for multiple non-parametric comparisons was used.

## 3. Results

The demographic, clinical, and laboratory characteristics of the study population are shown in Table 1. The COVID-19 group and the control group did not show statistically significant differences in demographic and clinical characteristics. The COVID-19 patients showed at admission statistically significant increased levels of fibrinogen (601.5 (480–747) vs. 455 (352.5–588.5) mg/dL; *p* = 0.0000064), and a higher percentage of patients had fibrinogen levels >400 mg/dL (86% vs.58%; *p* = 0.0054) compared to the control group.

**Figure 1:**
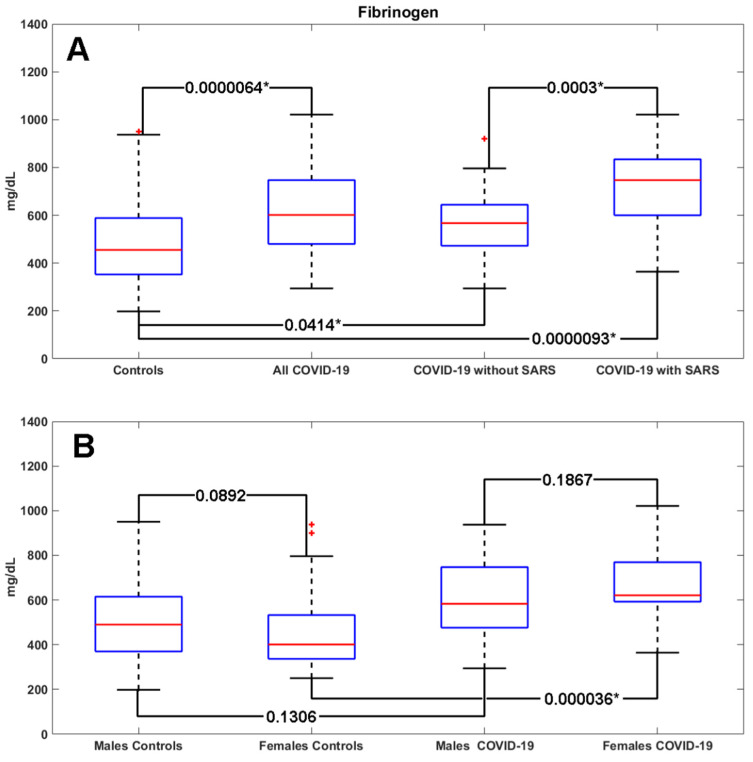
Fibrinogen values in the COVID-19 group versus the control group (**A**) and in COVID-19 patients with SARS vs. those without SARS (**B**). SARS: Severe Acute Respiratory Syndrome; * *p*-value < 0.05; red crosses: outliers.

When dividing the study population by gender, the values of fibrinogen appeared to be even higher in females affected by COVID-19 compared to females in the control group (*p* = 0.000036). A non-significant trend of increased fibrinogen levels was shown in COVID-19 male patients compared to male controls (Figure 1).

**Figure 2:**
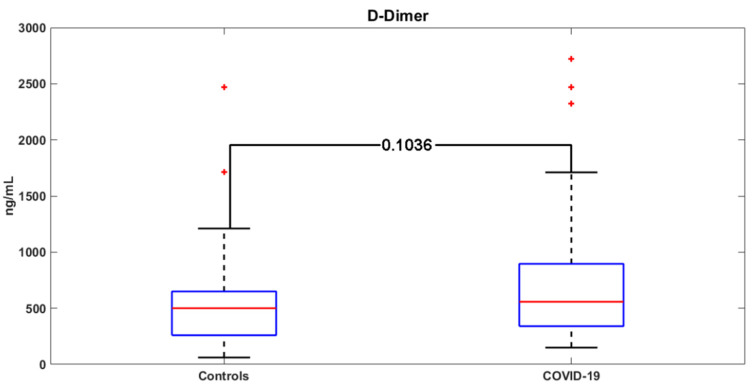
D-dimer values in the COVID-19 group versus the control group. Red crosses: outliers.

D-dimer levels were more frequently elevated in COVID-19 patients compared to controls (80% vs. 59%; *p* = 0.0946), without reaching statistical significance (Figure 2). No statistically significant difference in PLT, CRP, PT, and aPTT was found between COVID-19 patients and the control group (Table 2).

**Figure 3:**
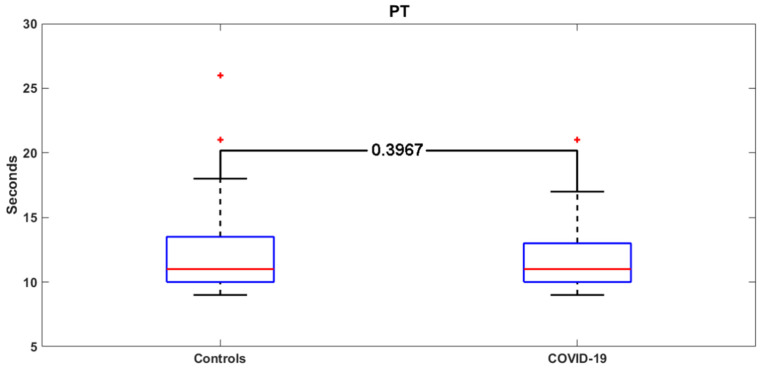
PT values in the COVID-19 group versus the control group. Red crosses: outliers.

**Figure 4:**
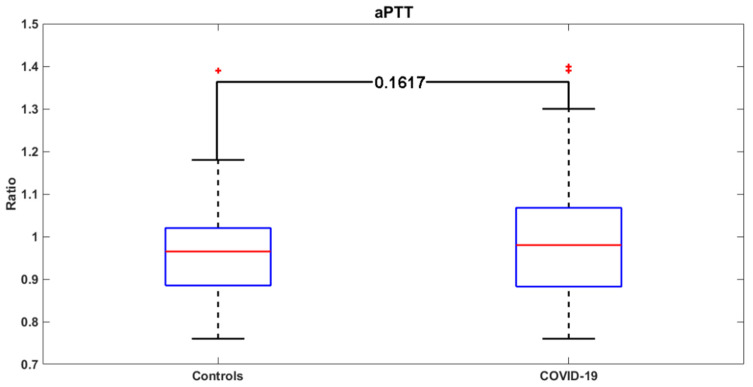
aPTT values in the COVID-19 group versus the control group. Red crosses: outliers.

Figure 3 and Figure 4 report the distribution of the PT and aPTT values in COVID-19 and control patients. The demographic, clinical, and laboratory characteristics of COVID-19 patients with and without SARS are shown in Table 3.

The COVID-19 patients with SARS were characterized at admission by statistically significant increased levels of fibrinogen (747 (600–834) vs. 567 (472.5–644.5) mg/dL; *p* = 0.0003) compared to patients without SARS; D-dimer levels showed a non-significant increasing trend (633 (484–2324) vs. 500 (281.7–740.5) mcg/dL; *p* = 0.075) in COVID-19 patients with SARS compared to those without SARS (Table 4).

## 4. Discussion

The association between alterations of hemostasis and infections is well known [7]. Bacterial infection, in particular those due to Gram-negative microorganisms, are able to activate the clotting system through both the release of tissue factor, with the following activation of the extrinsic pathway of the clotting cascade, and the induction of thrombin activation by the bacterial cell wall [8]. The hypercoagulable state, characterized by increased D-dimer levels, can occur since the early phase of bacterial infections and may lead to disseminated intravascular coagulation (DIC) [9]. On the other hand, viral infections may induce severe complications such as acute respiratory distress syndrome (ARDS) and multiorgan failure (MOF), which are two conditions frequently associated with hypercoagulation and DIC [10].

In the clinical setting of COVID-19, two recent studies described a SARS-CoV2-induced hypercoagulable state, characterized by relevant increases of D-dimer and Fibrin Degradation Products (FDP) [11], which can also be associated with a fatal outcome [6]. In particular, Zhou et al. evaluated the potential risk factors associated with poor prognosis in 191 COVID-19 patients and found that increased D-dimer levels was one of them; furthermore, Tang et al. showed that abnormal coagulation values, especially, markedly elevated D-dimer and FDP, were common in COVID-19 patients who did not survive [11].

Both the articles did not provide a clear relationship between COVID-19 clinical presentation and the alterations of hemostasis; moreover, they were focused exclusively on Chinese patients affected by COVID-19.

Up to date, no data are available about hemostasis alterations in non-Chinese COVID-19 patients and their relationship with the clinical presentation of the disease.

Our results from an Italian cohort of COVID-19 patients showed that clotting alterations, with a trend toward a hypercoagulable state due to increased levels of D-dimer and fibrinogen, were present at the early stage of the disease (i.e., at admission to the ED). Intriguingly, we found that increased levels of fibrinogen are more likely associated with a more severe form of COVID-19, characterized by SARS.

The hemostasis alterations described in our study population have been associated with an increased risk of thrombotic events or DIC in daily clinical practice [12,13]; moreover, abnormal values of fibrinogen and D-dimer have been previously described in several inflammatory, infectious, or other malignant diseases [12,13,14]; therefore, a clinical integrated method is always needed to evaluate the potential effects of this type of hypercoagulation [14].

Furthermore, since the first phases of COVID-19may be similar to those of other viral diseases such as flu, Zhang et al. suggested a specific triage selection for patients with suspected COVID-19, including the analysis of laboratory parameters which usually increase in the early stages of viral infection, such as C-reactive protein and lactate dehydrogenase (LDH) values [15], without the evaluation of the clotting system.

Concerning this clinical setting, our results showed that specific hemostasis alterations are already present in an early stage of SARS-CoV-2 infection, because our patients were analyzed at admission to the ED.

Since increased fibrinogen levels are more frequently shown in COVID-19 patients with SARS compared to COVID-19 patients without SARS, we hypothesize a possible association between the early increase of fibrinogen and the clinical form of COVID-19.

Since fibrinogen is not usually altered in the early phases of other viral infections, while it seems to increase early in COVID-19 patients, its activity should be routinely tested at ED triage of patients with fever and suspected COVID-19. We suggest its helpful role to early identify the COVID-19 subgroup at increased risk to develop SARS, which may benefit from a different and thorough clinical surveillance and treatment, because it is more frequently associated with high morbidity and mortality [2].

## 5. Conclusions

The routinely testing clotting factors at ED triage of patients with suspected SARS-CoV-2 infection could be useful to clinicians to collect more detailed information for the clinical management of COVID-19 patients. Although our data should be confirmed by studies on a larger population, fibrinogen seems to increase early in COVID-19 patients and may be used as a risk stratification marker for the early detection of a subgroup of COVID-19 patient at increased risk to develop SARS, who might benefit from a different and thorough clinical surveillance and treatment.

## Figures and Tables

**Table 1:** Demographic, clinical, and laboratory characteristics of the study population.

Patients’ Characteristics	COVID-19 Group *N*: 67	Control Group *N*: 67	*p*
Males, *n* (%)	47 (70%)	35 (52%)	0.09
Age <40 yy, *n* (%)	2 (3%)	3 (4%)	0.67
Age 40–60 yy, *n* (%)	19 (28%)	15 (22%)	0.74
Age >60 yy, *n* (%)	46 (68%)	49 (73%)	0.09
Cardiovascular diseases, *n* (%)	28 (41%)	25 (37%)	0.99
Concomitant Antiplatelets Drugs, *n* (%)	22 (32%)	30 (44%)	0.06
Concomitant Anticoagulants Drugs, *n* (%)	6 (9%)	10 (14%)	0.12
Asymptomatic Pneumonia, *n* (%)	2 (3%)	0 (0%)	0.24
Abnormal PT sec, *n* (%)	16 (23%)	7 (10%)	0.09
Abnormal aPTT, *n* (%)	2 (3%)	1 (2%)	0.73
D-dimer>500–700 mcg/dL, *n* (%)	54 (80%)	40 (59%)	0.09
Fibrinogen >400 mg/dL, *n* (%)	58 (86%)	39 (58%)	0.005

COVID-19: coronavirus disease 2019; PT: prothrombin time; aPTT: activated thromboplastin time.

**Table 2:** Comparisons of laboratory parameters between COVID-19 patients and controls.

Parameter	COVID-19 Group	Control Group	FPTest *p*-Value	FKTest *p*-Value
PT seconds	11(10.0–13.0)	11(10.0–13.5)	0.39	0.23
aPTT	0.96(0.85–1.07)	0.98(0.88–1.06)	0.16	0.08
PLT(mmcube)	360(244–413.5)	323(272–371)	0.28	0.13
CRP(mg/dL)	13.46(5.63–25.83)	11.00(6.00–25.00)	0.38	0.88
Fibrinogen (mg/dL)	622(448–796)	455(352.5–588.5)	0.0000064	0.71
D-dimer (mcg/dL)	556(327–859)	500(260 -650)	0.10	0.15

CRP: C-reactive protein; PLT: platelets; FP: Fligner–Policello; FK: Fligner–Killeen.

**Table 3:** Demographic, clinical, and laboratory characteristics of COVID-19 patients with and without SARS.

Patients’ Characteristics	COVID-19 with SARS *N*: 24	COVID-19 without SARS *N*: 43	*p*
Males, *n* (%)	15 (60%)	30 (69%)	0.73
Age <40 yy, *n* (%)	(0%)	2 (4%)	0.41
Age 40–60 yy, *n* (%)	11 (45%)	19 (44%)	0.99
Age >60 yy, *n* (%)	13 (54%)	24 (54%)	0.99
Cardiovascular diseases, *n* (%)	14 (58%)	14 (32%)	0.07
Concomitant Antiplatelets Drugs, *n* (%)	8 (33%)	14 (32%)	0.99
Concomitant Anticoagulants Drugs, *n* (%)	1 (4%)	2 (4%)	0.99
Abnormal PT, *n* (sec.)	8 33%)	9 (20%)	0.03
Abnormal aPTT, *n* (%)	0 (0%)	2 (4%)	0.73
D-dimer >500–700 mcg/dL, *n* (%)	21 (91%)	33 (76%)	0.41
Fibrinogen >400 mg/dL, *n* (%)	22 (92%)	35 (81%)	0.81

SARS: severe acute respiratory syndrome.

**Table 4:** Fibrinogen and D-dimer levels in COVID-19 patients with and without SARS.

Parameter	COVID-19 with SARS	COVID-19 without SARS	FP-Test *p*-Value	FK-Test *p*-Value
PT seconds	1.17(1.10–1.27)	1.15(1.10–1.22)	0.56	0.45
aPTT	0.92(0.81–1.12)	0.89(0.80–1.04)	0.14	0.62
PLT(mmcube)	320(210–595)	346(191–605)	0.32	0.15
CRP(mg/dL)	62(31–95)	55(28–93)	0.46	0.96
Fibrinogen (mg/dL)	747(600.0–834.0)	567(472.5–644.50)	0.0003	0.48
D-dimer (mcg/dL)	633(484–2324)	500(281.75–740.50)	0.075	0.21

Parameters are expressed as median and interquartile range. FP-test: Fligner-Policello test on medians; FK-test: Fligner-Killeen test on variances.
